# Supplementary material for: Structural basis of RNA processing by human mitochondrial RNase P
Source: Nat Struct Mol Biol. 2021 Sep 6;28(9):713–23. doi: 10.1038/s41594-021-00637-y (PMC8437803; doi:10.1038/s41594-021-00637-y)

nature portfolio

https://doi.org/10.1038/s41594-021-00637-y

Supplementary information

Structural basis of RNA processing by human mitochondrial RNase P

In the format provided by the authors and unedited

Supplementary Note 1

Table listing nucleotide positions in mitochondrial $tRNA^{Tyr}$ (tRNA numbering used in the deposited structural model) and corresponding canonical tRNA nucleotide positions (tRNA numbering used in the manuscript)

$tRNA^{Tyr}$	Canonical	Base	Region
1	1	G	Acceptor Stem
2	2	G	
3	3	U	
4	4	A	
5	5	A	
6	6	A	
7	7	A	
8	8	U	
9	9	G	
10	10	G	D Stem
11	11	C	
12	12	U	
13	13	G	
14	14	A	D Loop
15	16	G	
16	18	U	
17	20	G	
18	22	A	D Stem
19	23	A	
20	24	G	
21	25	C	
22	26	A	
23	27	U	Anticodon Stem
24	28	U	
25	29	G	
26	30	G	
27	31	A	
28	32	C	Anticodon Loop
29	33	U	
30	34	G	
31	35	U	
32	36	A	
33	37	A	
34	38	A	

$tRNA^{Tyr}$	Canonical	Base	Region
35	39	U	Anticodon Stem
36	40	C	
37	41	U	
38	42	A	
39	43	A	
40	44	A	Variable Region
41	45	G	
42	47	A	
43	48	C	
44	49	A	T Stem
45	50	G	
46	51	G	
47	52	G	
48	53	G	
49	54	U	T Loop
50	55	U	
51	57	A	
52	59	G	
53	60	G	
54	61	C	T Stem
55	62	C	
56	63	U	
57	64	C	
58	65	U	
59	66	U	Acceptor Stem
60	67	U	
61	68	U	
62	69	U	
63	70	A	
64	71	C	
65	72	C	
66	73	A	Discriminator Base

Supplementary Note 2

Sequence alignment of N-terminal region of TRMT10C orthologues

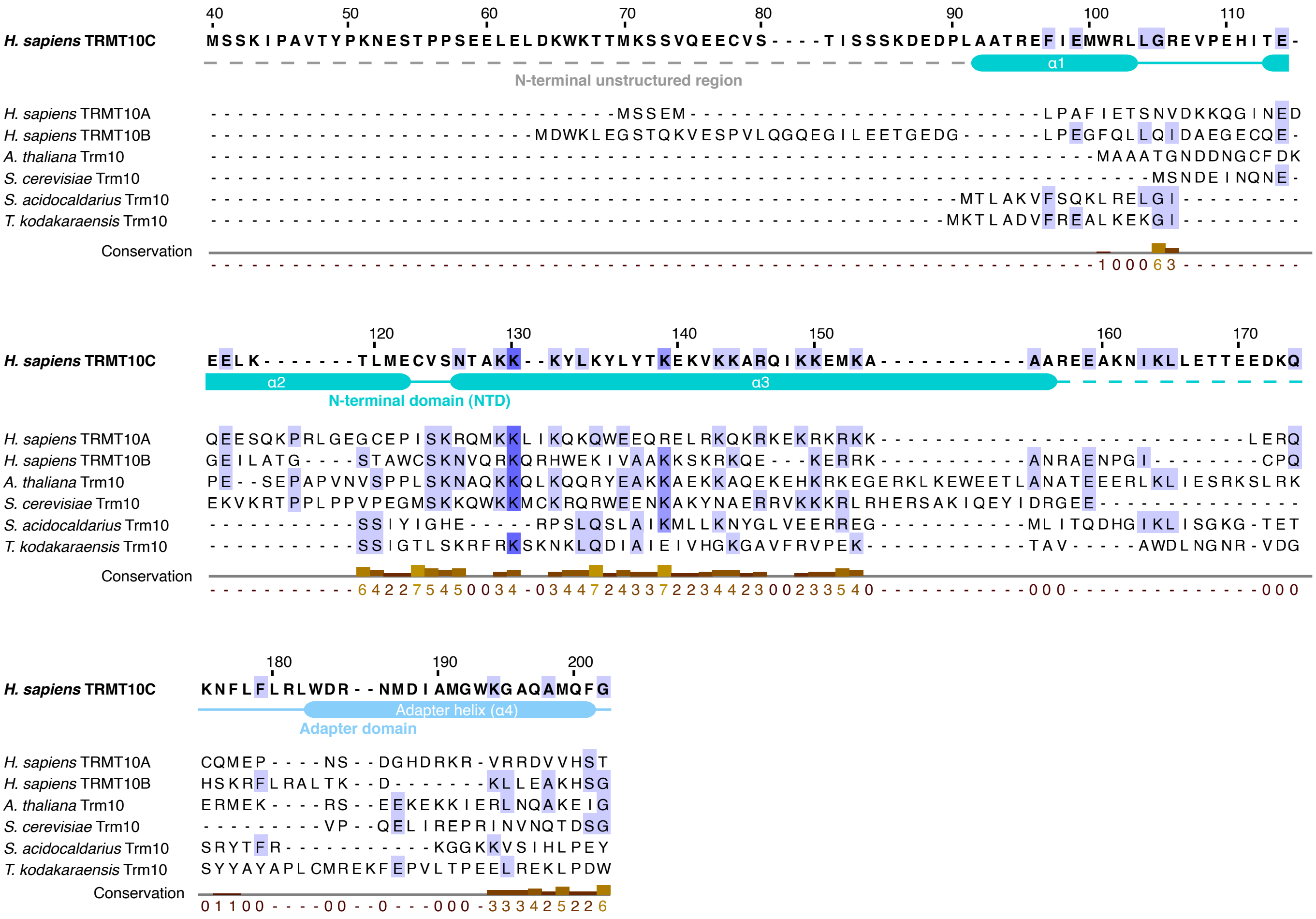

Supplementary Note 3

Sequence alignment of PPR domains of PRORP orthologues

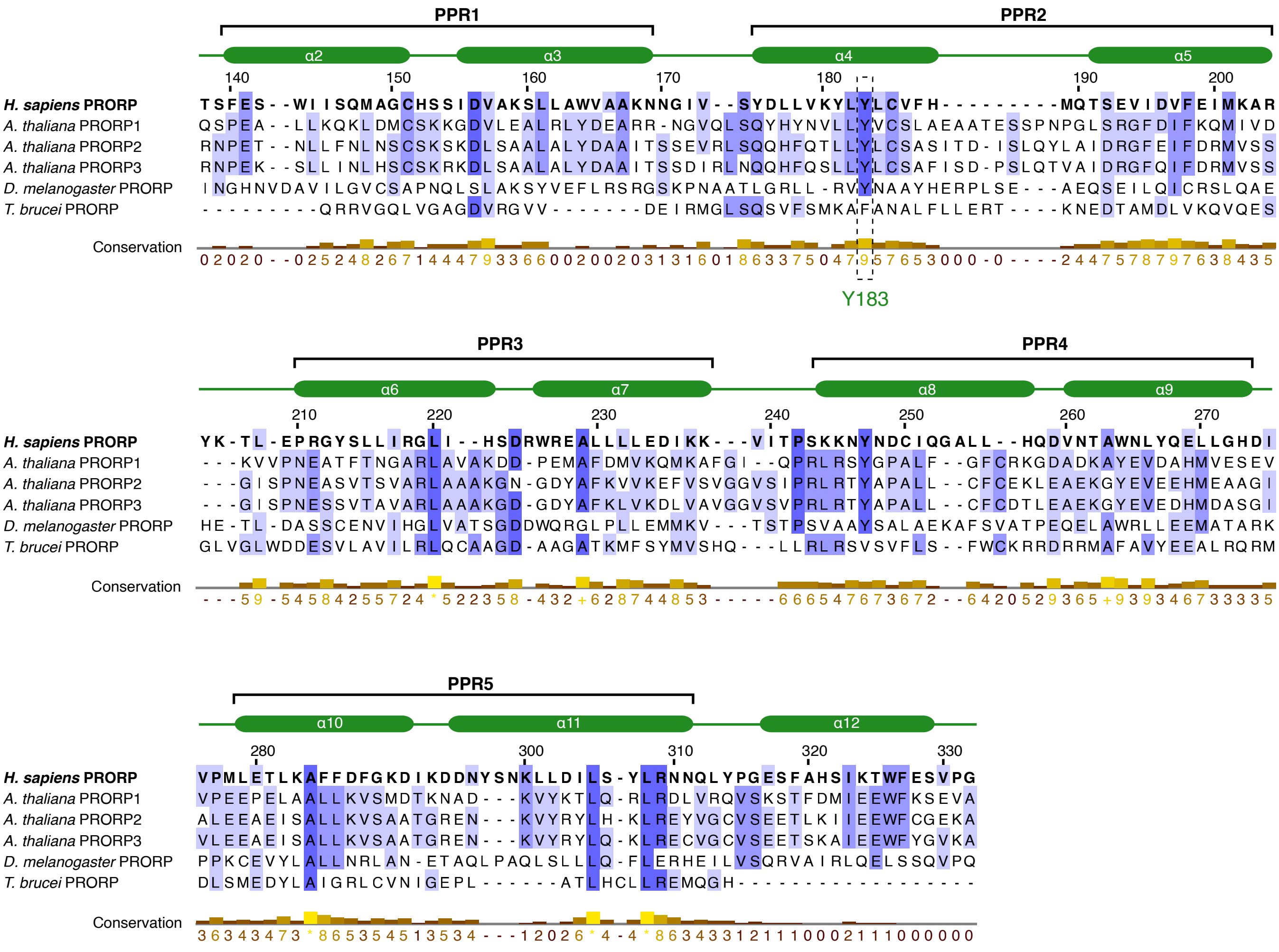

Supplement: Supplementary file 1 — Supplementary Notes 1–3. [file 41594_2021_637_MOESM1_ESM.pdf]
