# Supplementary material for: Structural basis of RNA processing by human mitochondrial RNase P
Source: Nat Struct Mol Biol. 2021 Sep 6;28(9):713–23. doi: 10.1038/s41594-021-00637-y (PMC8437803; doi:10.1038/s41594-021-00637-y)
Supplement: Source Data Extended Data Fig. 1 — Unprocessed images for gels shown in Extended Data Fig. 1 and gel images for replicates for panels b and c. [file 41594_2021_637_MOESM5_ESM.pdf]

Source Data Extended Data Figure 1

Source data for Extended Data Figure 1a
Dashed rectangle represents the area shown in Extended Data Figure 1a.

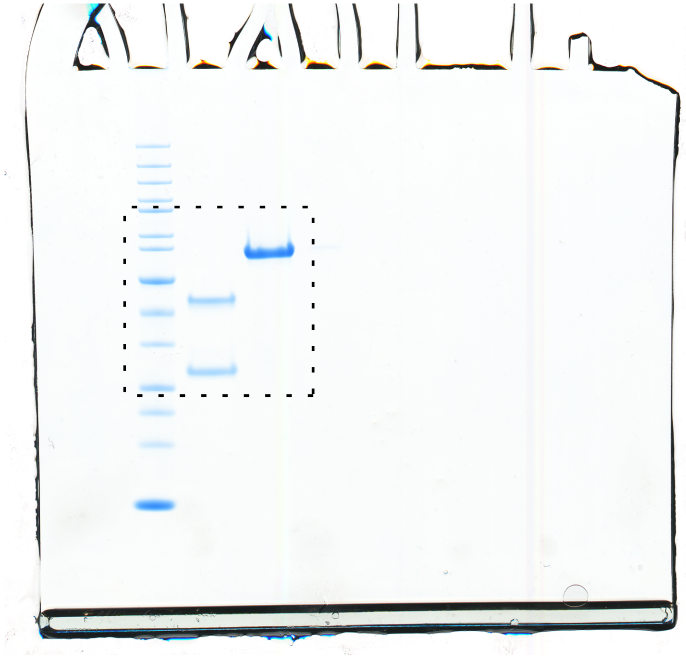

Molecular Weight Marker in kilodaltons (top to bottom)
200
150
120
100
85
70
60
50 (darker)
40
30
25
20
15
10 (darker)

Source data for Extended Data Figure 1b

RNA Size Marker in polynucleotide length (top to bottom)
1000, 800, 600, 400, 300, 200 (faint), 100

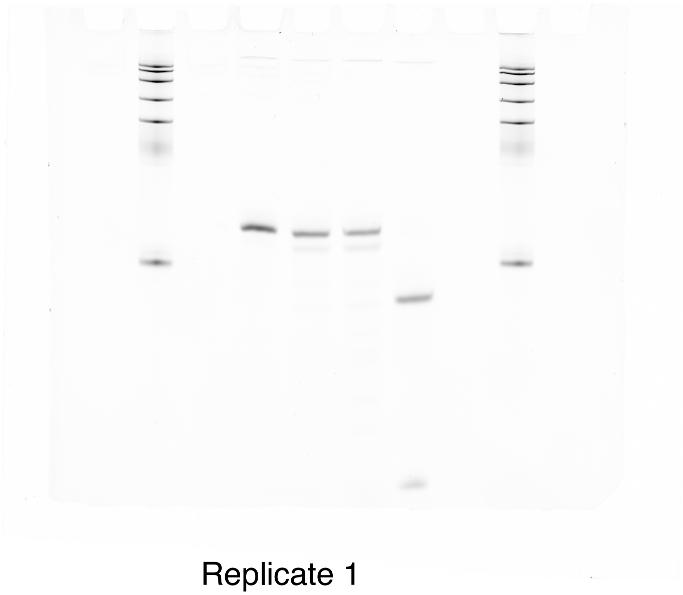

Replicate 1

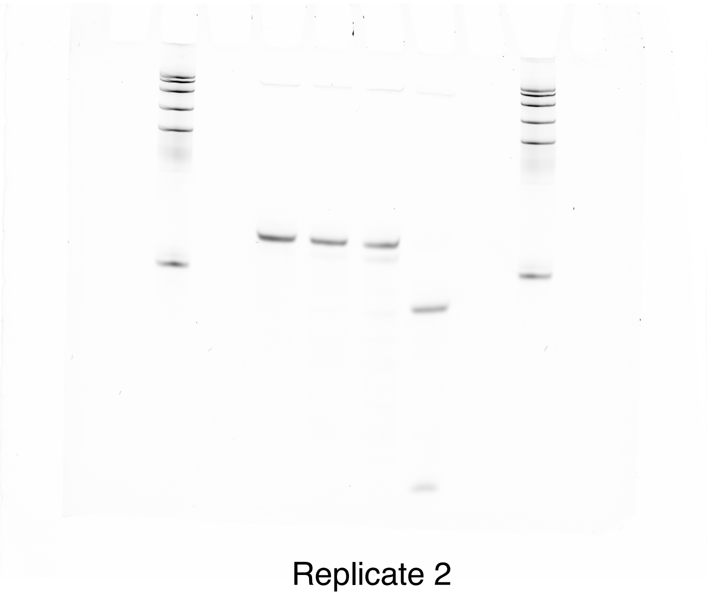

Replicate 2

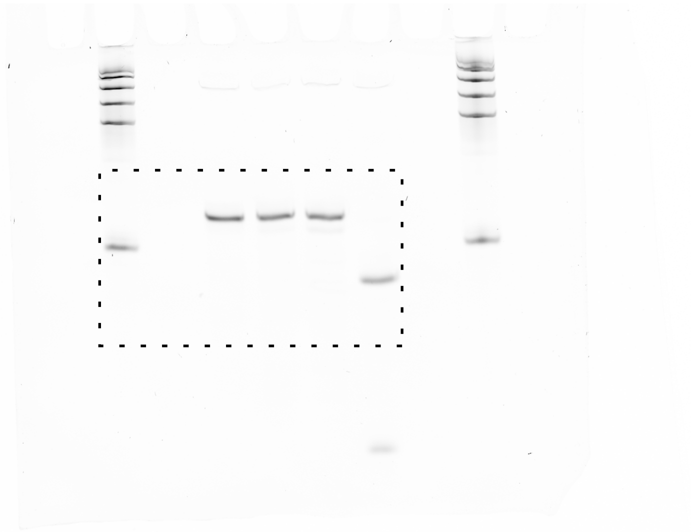

Replicate 3

Dashed rectangle represents
the area shown in Extended Data Figure 1b.

Source Data for Extended Data Figure 1c

RNA Size Marker in polynucleotide length (top to bottom)
1000, 800, 600, 400, 300, 200 (faint), 100

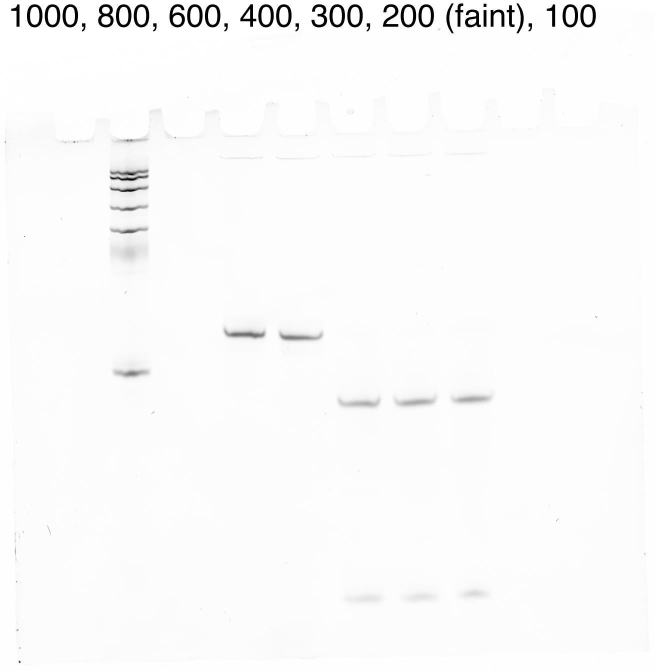

Replicate 1

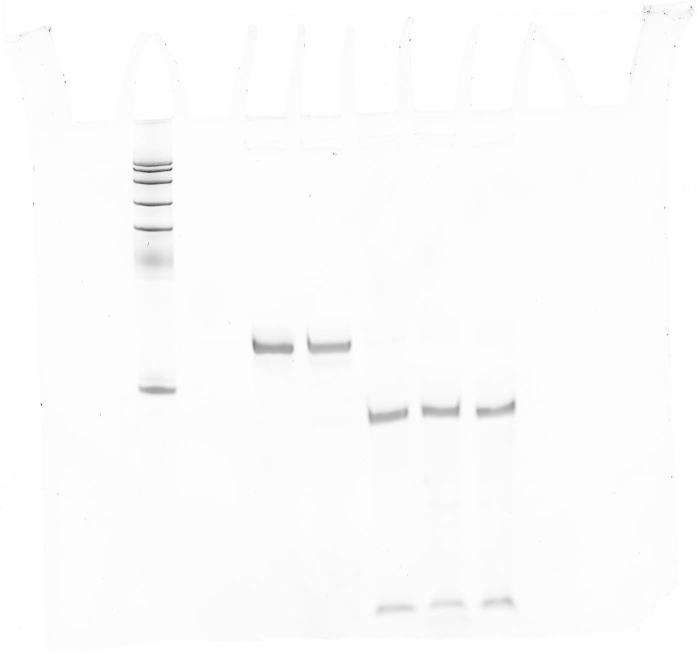

Replicate 2

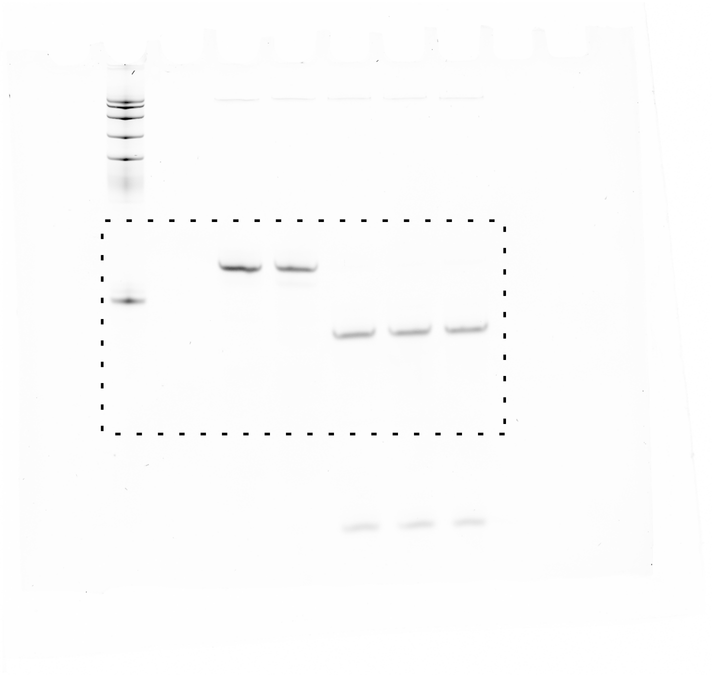

Replicate 3

Dashed rectangle represents
the area shown in Extended Data Figure 1c.

Source Data for Extended Data Figure 1d

Dashed rectangles represent the areas shown in Extended Data Figure 1d for protein (left) and RNA (right) gels.

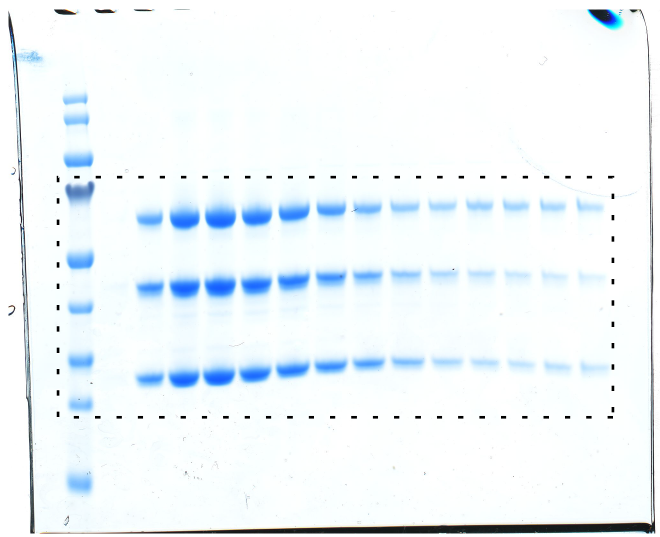

Molecular Weight Marker
in kilodaltons (top to bottom)
140
110
80
70 (darker)
50
40
30
25
15

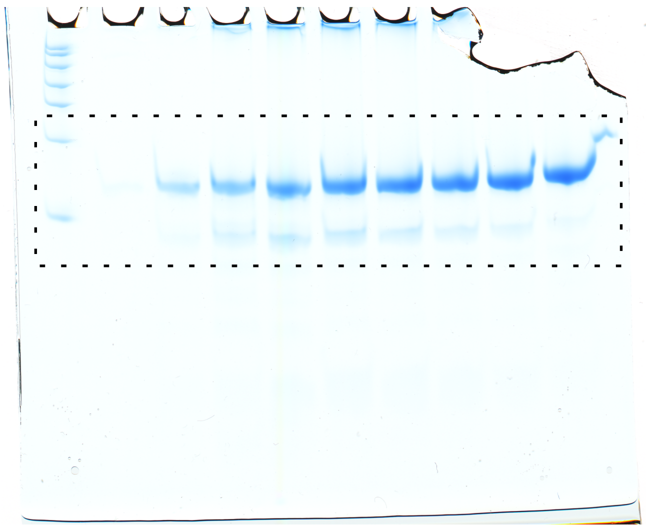

RNA Size Marker
in polynucleotide length
(top to bottom)
1000
800
600
400
300
200 (faint)
100
